# Persistence of the Effects of the COVID-19 Lockdown on Sleep: A Longitudinal Study

**DOI:** 10.3390/brainsci11111520

**Published:** 2021-11-17

**Authors:** Maurizio Gorgoni, Serena Scarpelli, Anastasia Mangiaruga, Valentina Alfonsi, Maria R. Bonsignore, Francesco Fanfulla, Luigi Ferini-Strambi, Lino Nobili, Giuseppe Plazzi, Luigi De Gennaro, on behalf of the Board of the Italian Association of Sleep Medicine (AIMS)

**Affiliations:** 1 Department of Psychology, Sapienza University of Rome, 00185 Rome, Italy; maurizio.gorgoni@uniroma1.it (M.G.); serena.scarpelli@uniroma1.it (S.S.); valentina.alfonsi@uniroma1.it (V.A.); 2 Department of Medical and Surgical Sciences, University of Bologna, 40126 Bologna, Italy; anastasiamangiaruga@gmail.com; 3 PROMISE Department, University of Palermo, and IRIB-CNR, 90127 Palermo, Italy; marisa.bonsignore@irb.cnr.it; 4 Sleep Medicine Unit, Clinical and Scientific Maugeri Institutes, Scientific Institute of Pavia, IRCCS, 27100 Pavia, Italy; ffanfulla@fsm.it; 5 Sleep Disorders Center, Vita-Salute San Raffaele University, 20127 Milan, Italy; ferinistrambi.luigi@hsr.it; 6 Child Neuropsychiatry Unit, IRCCS Istituto Giannina Gaslini, 16147 Genoa, Italy; lino.nobili@unige.it; 7 Department of Medical and Surgical Neuroscience and Rehabilitation (DINOGMI), University of Genoa, 16147 Genoa, Italy; 8 Department of Biomedical, Metabolic and Neural Sciences, University of Modena and Reggio Emilia, 41125 Modena, Italy; giuseppe.plazzi@unibo.it; 9 IRCCS Istituto delle Scienze Neurologiche di Bologna, 40139 Bologna, Italy; 10 Body and Action Lab, IRCCS Fondazione Santa Lucia, 00179 Rome, Italy

**Keywords:** COVID-19, pandemic, lockdown, sleep, stress, depression, insomnia, pre-sleep arousal

## Abstract

The effects of the COVID-19 pandemic on sleep have been widely documented, but longitudinal evaluations during different phases of the “COVID-19 era” are needed to disentangle the specific consequences of the r145estrictive measures on sleep variables. The aim of this study was to assess the immediate effect of the lockdown’s end on sleep and sleep-related dimensions in an Italian sample, also considering the stress and depressive symptoms. We used an online survey to longitudinally collect data on sociodemographic, environmental, clinical, sleep, and sleep-related variables in two time points: during and immediately after the lockdown. The final sample included 102 participants. The large prevalence of poor sleep quality, clinically relevant pre-sleep arousal, and depressive symptoms, as well as poor sleep quality and pre-sleep arousal score observed during the lockdown, remained stable after its end. On the other hand, the prevalence of moderate-to-severe event-related stress and intrusive symptom scores exhibited a drastic reduction after the end of home confinement. Both bedtime and rise time were anticipated after the lockdown, while sleep quality exhibited only a trend of post-lockdown sleep disturbance reduction. Our findings point to a reduced stress level (specific for the intrusive symptomatology) after the end of the lockdown and persistence of sleep problems, suggesting two non-mutually exclusive hypotheses: (a) the strict restrictive measures are not the main cause of sleep problems during the pandemic and (b) home confinement induces long-lasting effects on sleep observable after its end, and a longer period of time might be needed to observe an improvement.

## 1. Introduction

The dramatic effects of the COVID-19 pandemic on mental health have been widely documented [1,2]. Additionally, a large number of studies highlighted strong pandemic-related changes in sleep patterns [3,4,5,6,7,8,9,10].

It is hard to assess to what extent specific sleep changes are associated with specific consequences of the pandemic. Despite many cross-sectional studies providing evidence for the relation between specific sleep characteristics and different sociodemographic, COVID-19-related, and clinical measures, longitudinal studies are essential in this field. It is conceivable that the restrictive measures (e.g., quarantine) adopted to contain the spread of the virus may have had a strong impact on sleep habits and disturbances [11]. Indeed, home confinement can affect several areas of our daily experience (e.g., light exposure, social relationships, work schedules, physical activities, family management, use of electronic devices), which, in turn, are associated with changes in mood, stress levels, and sleep. In this view, an intriguing question is how sleep, together with overall mental health, changes during the different phases of the pandemic. Several studies longitudinally assessed sleep in large samples during different stages of the Italian lockdown, highlighting different time courses of sleep features according to several variables [12,13]. The same research group observed that the alarming prevalence of poor sleepers (~60%) and severe depression (~20%), widely documented during the first lockdown [4,6,12,14] persisted during the second wave [15]. In Austria, Pieh et al. [16] highlighted the long-lasting effects of the COVID-19 pandemic on depressive, anxiety, and insomnia symptoms after 6 months from the beginning of the lockdown, even though a slight improvement of stress and well-being was observed. Another Italian study highlighted a clear dissociation between changes in different sleep features 4 months after the end of the lockdown [9], suggesting that a longitudinal evaluation of sleep during different phases of the pandemic should be performed considering multiple sleep dimensions, with the aim to avoid simplistic generalizations.

Overall, these results indicate that the sleep pattern exhibits intense changes as a function of (a) the time spent in a specific condition (i.e., home confinement) and (b) modifications of the restrictive measures. Nevertheless, it remains unclear how long does it take to observe changes in sleep features after the end of the home confinement period. Indeed, the immediate effects of the end of the lockdown on sleep features have been substantially overlooked. Beck et al. [17] observed in France a reduced prevalence of sleep problems at the end of the confinement and confirmed this trend after one month. On the other hand, severe sleep problems associated with impairment of daily activities showed a more stable trend. However, sleep problems were assessed with only two items of the “Duke health profile” [18] in this study. Another research [19], aimed at confirming the effects of the lockdown on oneiric activity [14,20,21] using a longitudinal evaluation of daily sleep and dream diaries, found greater ease in falling asleep and reduced nocturnal awakenings, dream, and lucid dream recall frequency in an Italian sample during the first week after the confinement (compared with the last week of lockdown). However, possible parallel changes in emotional status were not measured.

The aim of this study was to assess the effect of the lockdown’s end on multiple sleep dimensions in an Italian sample, also considering changes in mental health status. Specifically, the novelty of the study is represented by the longitudinal evaluation of sleep quality, together with pre-sleep arousal, event-related stress, and depression, during the last period of the Italian lockdown and after its end.

## 2. Materials and Methods

### 2.1. Design and Participants

The Italian lockdown implemented to contain the first wave of COVID-19 started on 9 March 2020 and ended on 4 May 2020, and it consisted of home confinement and social distancing. It was followed by a period of reduced restrictive measures that lasted until Fall 2020, when regional restrictive measures to contain the second wave of contagion were implemented. This longitudinal study was designed by the Italian Association of Sleep Medicine (Associazione Italiana di Medicina del Sonno—AIMS) board to assess sleep, stress, and depression during the last period of the first lockdown (T1: 1 April 2020–4 May 2020) and immediately afterward (T2: 8 May 2020–7 June 2020). The mean time interval (±standard deviation) for answering the questionnaires at T1 and T2 was 33.31 ± 5.63 days. During both planned periods, we shared an online survey on several social media (Facebook, Twitter, Instagram, the AIMS website), collecting data from Italian participants with age ≥ 18 years. The same questionnaire was used at T1 and T2. At any moment, the participant could withdraw from the survey without their data being saved. We did not provide monetary compensation. Each participant explicitly agreed to take part in the research after reading an informed consent. The study was approved by the Institutional Review Board of the Department of Psychology, Sapienza University of Rome (#0000585, 31 March 2020) and conducted in accordance with the Declaration of Helsinki.

**Figure 1:**
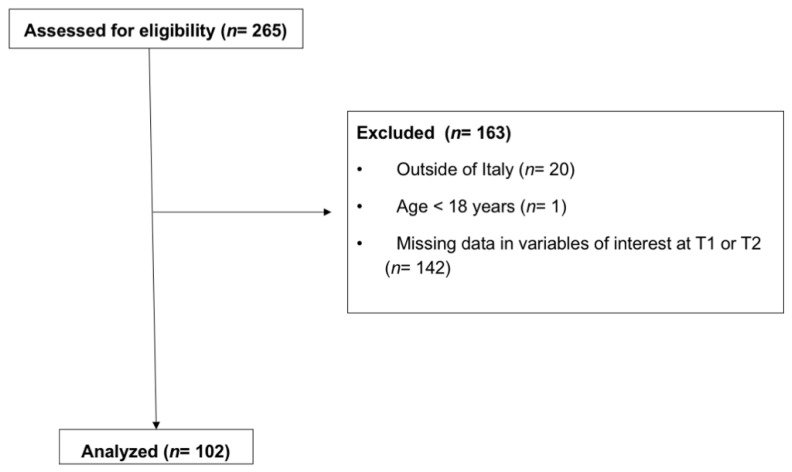
Description of the participants’ enrollment.

Overall, 265 participants compiled the questionnaire at both T1 and T2. Exclusion criteria were living outside of Italy at T1 and/or T2; participants infected by the COVID-19; age < 18 years; the presence of missing data in variables of interest at T1 or T2 (Figure 1). No participant in the original sample was infected by the COVID-19. The final sample considered for the analyses included 102 participants.

### 2.2. Materials

Sociodemographic and COVID-19-related data: we collected information about gender, age, education, work, Italian region, having a partner, having children, cohabitation, having a relative/friend infected by COVID-19, home size, and current intake of medications. We also collected the number of hours per day spent at home before, during, and after the lockdown. Time spent at home was the only pre-lockdown measure collected.

Stress: we used the Italian version of the Impact of Event Scale (IES) [22], initially developed by Horowitz [23], to investigate self-reported event-related traumatic stress through 15 items. Seven items measure intrusive symptoms, while eight items assess avoidance. Overall, a moderate-to-severe stressful impact is indicated by a total score ≥ 26. We asked the participants to refer to any traumatic event occurring during the last week and associated with the pandemic. The participant’s compliance was assessed controlling the pandemic-related content of the reported event.

Depressive symptoms: we administered the Beck Depression Inventory (BDI-II, [24]), a self-reported questionnaire (21 items), to assess depressive symptoms. A total score > 13 suggests the presence of a depressive disorder.

Sleep quality: sleep quality was assessed by the Italian version of the Pittsburgh Sleep Quality Index (PSQI—[25]). It is a self-reported questionnaire consisting of 19 items, resulting in 7 sub-scales (sleep quality, sleep latency, sleep duration, habitual sleep efficiency, sleep disturbances, use of sleep medications, daytime dysfunction) and a sleep quality global score. A PSQI global score > 5 points to poor subjectively perceived sleep quality. Beyond these measures, we also extracted specific sleep variables from the PSQI: total bedtime (TBT, min), total sleep time (TST, min), bedtime (24 h clock), and rise time (24 h clock).

Pre-sleep arousal: we administered the Italian adaptation of the Pre-Sleep Arousal Scale (PSAS—see [26]) to investigate pre-sleep arousal [27]. It consists of 16 items for the assessment of self-reported cognitive (8 items) and somatic (8 items) arousal perceived while attempting to fall asleep. We used pathological cut-off scores of ≥14 and ≥20 for the somatic and cognitive subscale, respectively [28]. In a previous study, we found that almost half of the considered sample suffered from clinically relevant levels of at least one component of pre-sleep arousal during the Italian lockdown [29].

Circadian preference: the Italian reduced version of the Morningness–Eveningness Questionnaire (rMEQ—[30]) was used to assess the individual chronotype. It is a self-reported questionnaire consisting of five items. Its score ranges between 4 and 25: higher scores point to morningness preference. According to the cut-off criteria, we divided participants into evening-type, neutral-type, and morning-type groups.

### 2.3. Statistics

The analyses were run in JAMOVI 1.6 (The Jamovi project, 2020), Statistical Package for the Social Sciences (SPSS, version 27, IBM SPSS, Chicago, IL, USA), and MATLAB R2011b. We performed descriptive analyses to outline sociodemographic, COVID-19-related, clinical, and sleep measures of the sample. Variables were presented as absolute (*n*) and relative (%) frequency. The mean and standard error (SE) were reported for continuous variables.

A repeated-measures ANOVA was performed on the h/d spent at home (pre-lockdown, lockdown, post-lockdown). LSD method was used for post hoc comparisons.

An exact McNemar test was performed to assess differences between the two time points in the proportion of absence of work, presence of cohabitation, presence of relatives/friends with COVID-19, morning-/neutral-/evening-type, poor sleep quality, high pre-sleep arousal, presence of depressive symptoms, and event-related stress. We also reported positive predictive value (i.e., the likelihood of an individual with a score above the cut-off at T1 to exhibit a score above the cut-off at T2) and negative predictive value (i.e., the likelihood of an individual with a score below the cut-off at T1 to exhibit a score below the cut-off at T2) for the main sleep and clinical measures considered.

One-way repeated-measures ANOVAs were performed to assess changes in IES, BDI, rMEQ, and PSQI global score during and after the lockdown. To assess longitudinal changes in IES, PSQI, and PSAS subscales and PSQI additional measures we performed for each test a one-way repeated-measures MANOVA using “time” (T1 vs. T2) as a within-subject factor. To understand which conditions were different at the univariate level, we performed univariate ANOVAs. Before applying the above tests, the assumption of normality was checked: only two subscales of the PSQI (i.e., habitual sleep efficiency and sleep medication) violated this assumption. However, starting from the robustness of ANOVA and MANOVA to some violation of normality, we preferred to not correct the data in order to perform the MANOVAs.

## 3. Results

### 3.1. Sociodemographic, Sleep, and Clinical Characteristics of the Sample

Sociodemographic features are reported in Table 1.

**Figure 2:**
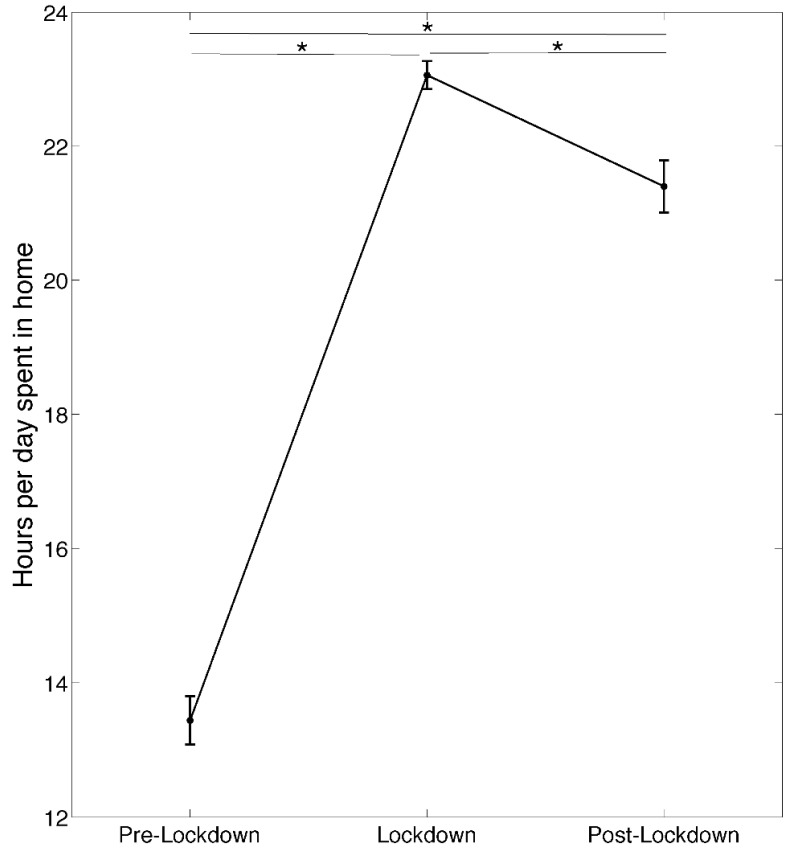
Results of the repeated-measures ANOVA performed on the hours per day spent at home in three time points: pre-lockdown, lockdown, and post-lockdown. Asterisks and thin black lines represent significant differences (*p* < 0.05) between time points after LSD post hoc comparison.

Figure 2 depicts the results of the repeated-measures ANOVA conducted on h/d spent at home. We found a significant difference (F_2,198_ = 281.20; *p* < 0.00001) between the considered time points. Post hoc LSD revealed that both lockdown (*p* < 0.00001) and post-lockdown (*p* < 0.00001) were characterized by a greater number of h/d spent at home compared to the pre-lockdown period. Moreover, the post-lockdown period exhibited reduced h/d spent at home compared with the lockdown (*p* < 0.00001).

Table 2 reports the proportion of subjects at T1 and T2 divided according to COVID-19-related, sleep, and clinical measures. About half of the sample worked during (53.92%) and after (49.02%) the lockdown and knew relatives/friends infected by COVID-19 (T1: 47.06%; T2: 55.88%). The larger part of the participants lived with other people at both time points (T1: 77.45%; T2: 80.39%). For what concerns sleep and sleep-related features, about half of the sample had a neutral chronotype (T1: 50.00%; T2: 45.10%), with the other half was almost equally divided between evening (T1: 23.53%; T2: 29.41%) and morning (T1: 26.47%; T2: 25.49%) types. The majority of the participants exhibited poor sleep quality both during (61.76%) and after (59.80%) the lockdown. Moreover, almost half of the sample showed clinically relevant pre-sleep arousal in the cognitive (T1: 49.02%; T2: 45.10%) and somatic (T1: 40.20%; T2: 41.18%) domains in both time points.

About 30% of the sample reported depressive symptoms (T1: 30.39%; T2: 36.27%), and moderate-to-severe event-related stress was observable in 73.53% and 55.88% of the sample during and after the lockdown, respectively. Almost half of the sample used medications (T1: 49.02%; T2: 46.08%).

The direct assessment (McNemar test) of post-lockdown changes in the distribution of participants according to COVID-19-related, sleep, and clinical measures revealed a significant difference (*p* = 0.001) only in event-related traumatic stress (IES) scores. In particular, 24 participants passed from a high level of event-related stress during the lockdown to a low level of event-related stress after the end of the home confinement period, while only 6 participants exhibited an opposite direction of changes.

Positive predictive value and negative predictive value for sleep, sleep-related, and clinical measures are reported in Table 3.

### 3.2. Post-Lockdown Changes in Clinical and Sleep Measures

**Figure 3:**
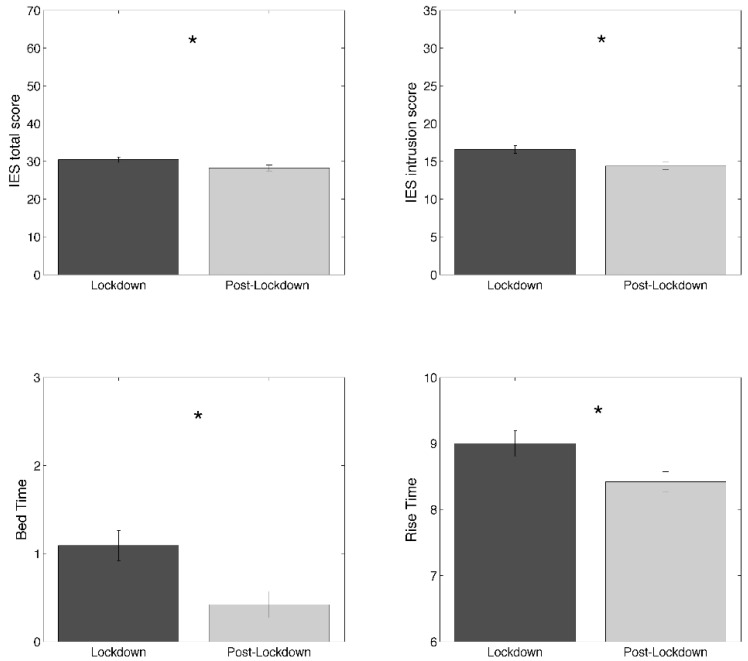
Clinical and sleep variables showing significant results (asterisked; *p* < 0.05) of the lockdown vs. post-lockdown comparisons (univariate ANOVAs): Impact of Event Scale (IES) total score; IES intrusion score; bedtime; rise time. Clinical and sleep variables showing significant results (asterisked; *p* < 0.05).

The direct comparisons (ANOVAs) between T1 and T2 concerning the scores obtained in the global scores of sleep and clinical tests are reported in Table 4, showing a significant reduction in event-related stress (F_1,101_ = −3.79; *p* = 0.0003) after the lockdown without changes in BDI, rMEQ and PSQI global score. One-way MANOVAs conducted on the subscales of sleep and clinical tests showed a statistically significant difference for IES subscales (Wilks’ λ = 0.75, F_2,100_ = 16.44, *p* < 0.00001), PSQI subscales (Wilks’ λ = 0.85, F_7,95_ = 2.38, *p* = 0.027) and PSQI additional measures (Wilks’ λ = 0.92, F_3,99_ = 2.85, *p* = 0.041), without significant changes concerning PSAS subscales (Wilks’ λ = 0.98, F_2,100_ = 0.75, *p* = 0.47). The results of the ANOVAs performed to understand which conditions were different at the univariate level are reported in Table 4. The analyses conducted separately for the intrusion and the avoidance components of the IES score revealed that the post-lockdown reduction was specific for the intrusive symptoms (F_1,101_ = 31.58; *p* < 0.00001). Considering PSQI subscales, we found no significant changes, albeit a trend (F_1,101_ = 3.90; *p* = 0.051) of post-lockdown reduced sleep disturbance was observed. We found a statistically significant anticipated bed time (F_1,101_ = 6.10; *p* = 0.02) and rise time (F_1,101_ = 5.30; *p* = 0.02) after the lockdown. Finally, no difference characterizes chronotype and pre-sleep arousal measures. The observed significant differences in the ANOVAs are summarized in Figure 3. It is worth noting that IES, PSQI, PSAS-Cognitive, and PSAS-Somatic mean scores were above the cut-off level for moderate-to-severe stress, poor sleep quality, and high pre-sleep arousal both during and after the lockdown.

## 4. Discussion

This study was designed to assess the effects of the first Italian lockdown’s end on sleep, stress, and depression symptoms during the COVID-19 pandemic. The main findings highlight that (a) the high percentage of participants with poor sleep quality, clinically relevant pre-sleep arousal, and depressive symptoms observed during the lockdown remained substantially stable after its end; (b) the prevalence of moderate-to-severe event-related stress and the intrusive event-related stress symptoms scores were drastically reduced after the end of the confinement; (c) the post-lockdown period was characterized by anticipation of both bedtime and rise time; (d) beyond a trend of reduced sleep disturbance after the end of the confinement, poor sleep quality and clinically relevant pre-sleep arousal observed during the lockdown remained stable after its end; (e) the time spent at home in the post-lockdown period was reduced compared with the lockdown, but both periods were characterized by greater h/d spent at home compared with the pre-lockdown period.

Considering sleep, the only significant difference concerned the anticipation of bedtime and rise time after the lockdown. Several findings highlighted that the lockdown period was characterized by later bedtime and rise time [5,31,32,33]. The more flexible working and social schedules during the home confinement (associated with smart working and homeschooling) may have offered the opportunity to set sleep timing according to individual circadian preferences [34]. In this view, it is conceivable that the end of home confinement implies a reorganization of daily habits under a greater influence of social zeitgebers (e.g., return to workplace and school), with consequent anticipation of habitual bedtime and rise time. Anticipation of the rise time has been also observed four months after the end of the confinement, compared with lockdown habits [9].

Our findings suggest that the end of the lockdown did not involve immediate significant changes in sleep quality, duration, and pre-sleep arousal. In particular, we confirmed an alarming prevalence of poor sleep quality and pre-sleep arousal during the lockdown [3,4,5,6,12,14,29,35], which remained high after the home confinement period. Only a trend of post-lockdown sleep disturbance reduction was observed, which is substantially in line with the few previous results collected immediately after the lockdown in the direction of a sleep improvement [17,19]. This result suggests that the end of home confinement had only a slight positive effect on sleep disturbance in the short term, while long-lasting detrimental effects of the lockdown on sleep may need a longer time to exhibit a substantial improvement. It should be considered that even though the lockdown ended, we continued to experience the pandemic, with its environmental and emotional consequences. Moreover, many restrictive measures in Italy have been eased only progressively during the spring–summer period of 2020. Consistently, our results showed that the time spent at home after the lockdown was reduced, compared with the home confinement period, but it was significantly greater than the pre-lockdown period, suggesting that participants in our sample did not return to the habitual pre-pandemic daytime schedule immediately after the lockdown. It is worth noting that an Italian study found substantially stable sleep patterns during the lockdown through a week-by-week assessment of sleep diaries, followed by a reduction in sleep onset latency, time in bed, and dream frequency and an increase in the perceived ease to fall asleep 4 months after the end of the lockdown [36]. Therefore, it is possible that a longer period of time in a condition of relative stability and a further reduction in the restrictions are needed to observe major changes in sleep quality after the end of home confinement.

In this view, our results concerning emotional status appear of particular interest. It was confirmed that depressive and event-related stress symptoms were highly prevalent during the lockdown period in Italy [4,5,6,14,15]. However, while mean depression score and prevalence remain stable after the lockdown, results on stress appear more complex. On the one hand, we observed a strong post-lockdown reduction in stress levels, mirrored by a decreased prevalence of participants reporting moderate-to-severe event-related stress. On the other hand, the mean stress score remains in a moderate range after the lockdown, and the reduction in stress levels was specific for the intrusive symptoms, while it did not involve the avoidance measure. The longitudinal assessment of the psychological impact of the lockdown highlighted heterogeneous results [37], and the differences in the specific phases of the pandemic when data were collected should be carefully considered. A Chinese study performed during the first weeks of confinement highlighted the negative psychological impact of the lockdown compared with a previous period [38]. Another study conducted in China at the beginning of the outbreak and after 4 weeks (when the number of daily COVID-19 cases was reduced) highlighted a reduction in post-traumatic stress disorder symptomatology in the second survey, without differences in anxiety, depression, and stress [39]. An increase in psychological distress has been observed at the initial phases of the lockdown in the US, followed by a reduction after several months [40]. Similarly, an increase in mental health problems has been observed during the lockdown, compared with pre-pandemic data, in the UK, followed by a reduction during a period with eased restrictions [41]. However, mental health problems remain significant, compared with the pre-pandemic condition [41]. Finally, an Austrian study assessing depression, anxiety, sleep quality, perceived stress, quality of life, and well-being found that the negative consequences of the pandemic persisted for several months after the end of the lockdown, with only a slight improvement in stress and well-being measures [16]. Our present findings are consistent with those observations pointing to a reduction in the stress level after the end of the lockdown and persistence of sleep and mental health problems, suggesting the following two non-mutually exclusive hypotheses: (a) the strict restrictive measures are not the main cause of sleep problems during the pandemic and (b) home confinement induces long-lasting effects on sleep observable also after its end. Moreover, our results highlight a clear dissociation between the intrusive and avoidance symptoms. On one hand, the post-lockdown reduction in intrusions points to a negative impact of home confinement on this kind of stress symptoms. On the other hand, the absence of differences in the avoidance score suggests that other factors rather than home confinement may be specifically associated with avoidance symptomatology.

Several limitations should be considered. First, as observed in previous papers (e.g., [14]), the online recruitment strategy may introduce a significant bias, attracting a larger number of participants with sleep or mental health problems. Moreover, our sample cannot be considered representative of the Italian population, being unbalanced for several biases. In particular, we had a greater prevalence of women (83.33%), which is a common condition of many surveys conducted during the pandemic (e.g., [4,5,14,19,20]).

Another limitation is represented by the smaller sample size of our study compared with other COVID-19 online surveys. It is possible that the compilation of the same long questionnaire after a brief period of time from the first survey may have discouraged many individuals from participating. Moreover, it is worth noting that we did not send any reminders to participants after contacting them for the second survey. Finally, we excluded a large number of participants because several variables of interest were missing at T1 or T2.

We cannot provide information about the pre-pandemic sleep and mental health condition, limiting the possibility to draw conclusions about the effect of the lockdown. It is also worth noting that we did not collect objective measures of sleep. Finally, we should consider the possibility that those subjects with improved sleep quality after the lockdown may have had reduced interest in the questionnaire and neglected to participate in the second survey. This could have an impact on the absence of significant sleep changes found in the study, but we do not have the possibility to control for this issue.

## 5. Conclusions

In this paper, we observed that the alarming prevalence of individuals with poor sleep quality, clinically relevant pre-sleep arousal, and depressive symptoms remained high after the lockdown. Beyond anticipation of sleep time, the end of home confinement during the COVID-19 pandemic had only a slight (positive) effect on sleep. Additionally, we found a selective reduction in intrusive stress symptoms after the lockdown, without changes in avoidance and depressive symptomatology. Understanding sleep and mental health oscillations during the different phases of the pandemic is essential from a clinical standpoint. The fight against COVID-19 is not over, and the observation of long-lasting sleep and mental health problems after the lockdown means that politicians and clinics should not let down their guard on citizens’ psychological well-being after the end of the home confinement periods and should provide adequate measures to counteract sleep and psychological problems during the course of the pandemic.

## Figures and Tables

**Table 1:** Demographic characteristics of the sample.

	Overall Sample (*n* = 102)	
	N	%
Demographic features		
Gender		
Male	17	16.67
Female	85	83.33
Age (Mean ± SE: 36.69 ± 1.50 years)		
18–25	35	34.31
26–30	15	14.71
31–40	15	14.71
41–50	13	12.74
>50	24	23.53
Education		
Middle school	1	0.98
High school	36	35.29
Undergraduate/Graduate	62	60.78
Post-graduate	3	2.94
Italian area		
North	43	42.15
Center	46	45.09
South	13	12.75
Having a partner		
No	41	40.20
Yes	61	59.80
Having children		
No	78	76.47
Yes	24	23.53
Home size (Mean ± SE: 111.00 ± 6.27 sq. m.)		

**Table 2:** Proportion of participants concerning COVID-19-related, sleep, and clinical variables during (T1) and after (T2) the lockdown. Results (*p*-values) of the McNemar tests were also reported. Asterisks mark significant differences (*p* < 0.05).

	Lockdown (T1) ^a^		Post-lockdown (T2) ^b^		*p*
	N	%	N	%	
*COVID-19-related features*					
Work					0.27
No	47	46.08	52	50.98	
Yes	55	53.92	50	49.02	
Cohabitation					0.37
No	23	22.55	20	19.61	
Yes	79	77.45	82	80.39	
Knowing a relative/friend infected by COVID-19					0.08
No	54	52.94	45	44.12	
Yes	48	47.06	57	55.88	
*Sleep and clinical features*					
rMEQ					0.27
Evening type	24	23.53	30	29.41	
Neutral type	51	50.00	46	45.10	
Morning type	27	26.47	26	25.49	
PSQI Global					0.85
PSQI ≤ 5	39	38.24	41	40.20	
PSQI > 5	63	61.76	61	59.80	
PSAS-Cognitive					0.54
PSAS-C ≤ 19	52	50.98	56	54.90	
PSAS-C > 19	50	49.02	46	45.10	
PSAS-Somatic					1.00
PSAS-S ≤ 13	61	59.80	60	58.82	
PSAS-S > 13	41	40.20	42	41.18	
BDI					0.24
BDI ≤ 13	71	69.61	65	63.73	
BDI > 13	31	30.39	37	36.27	
IES					* 0.001
IES ≤ 25	27	26.47	45	44.12	
IES > 25	75	73.53	57	55.88	
Current intake of medication					0.45
No	52	50.98	55	53.92	
Yes	50	49.02	47	46.08	

^a^ T1: 1 April 2020–4 May 2020; ^b^ T2: 8 May 2020–7 June 2020.

**Table 3:** Positive predictive value and negative predictive value for IES, BDI, PSQI, and PSAS.

	Positive Predictive Value	Negative Predictive Value
IES	68%	77.8%
BDI	80.6%	83.1%
PSQI	76.2%	66.7%
PSAS-C	72%	80.8%
PSAS-S	68.3%	77%

**Table 4:** Results of the comparisons (univariate ANOVAs) between lockdown (T1) and post-lockdown (T2) clinical, sleep, and sleep-related measures. Mean and standard errors (SE) are reported for the two time points. Asterisks index significant differences (*p* < 0.05).

	Lockdown (T1) ^a^ Mean ± SE	Post-Lockdown (T2) ^b^ Mean ± SE	F_1,101_	*p*
IES total score	30.43 ± 0.73	28.25 ± 0.80	14.34	* 0.0003
IES Intrusion	16.57 ± 0.51	14.38 ± 0.51	31.58	* <0.00001
IES Avoidance	13.86 ± 0.38	13.87 ± 0.41	0.001	0.98
BDI	11.73 ± 0.89	12.08 ± 0.90	0.24	0.62
rMEQ	14.43 ± 0.39	14.18 ± 0.40	1.52	0.22
PSQI—Global Score	6.75 ± 0.36	6.75 ± 0.34	0.00	1.00
PSQI C1—Subjective sleep quality	1.52 ± 0.09	1.41 ± 0.08	1.88	0.17
PSQI C2—Sleep latency	1.57 ± 0.12	1.44 ± 0.11	2.35	0.13
PSQI C3—Sleep duration	0.75 ± 0.07	0.83 ± 0.07	1.53	0.22
PSQI C4—Habitual sleep efficiency	0.33 ± 0.08	0.50 ± 0.09	2.98	0.09
PSQI C5—Sleep disturbance	1.35 ± 0.05	1.23 ± 0.05	3.90	0.051
PSQI C6—Sleeping medication	0.42 ± 0.10	0.46 ± 0.10	0.35	0.56
PSQI C7—Daytime dysfunctions	0.80 ± 0.07	0.87 ± 0.07	1.09	0.30
Total bedtime (min)	471.75 ± 8.73	480.06 ± 8.15	0.74	0.39
Total sleep time (min)	424.41 ± 7.93	418.14 ± 7.61	1.03	0.31
Bed time	01.09 ± 0.17	00.42 ± 0.15	6.10	* 0.02
Rise time	09.00 ± 0.19	8.42 ± 0.15	5.30	* 0.02

^a^ T1: 1 April 2020–4 May 2020; ^b^ T2: 8 May 2020–7 June 2020.

## Data Availability

The data presented in this study are available on request to the corresponding author.
